# Impact of preoperative bowel preparation methods on anastomotic leakage and intestinal motility recovery in laparoscope-assisted heart-shaped anastomosis

**DOI:** 10.3389/fsurg.2025.1554493

**Published:** 2025-10-03

**Authors:** Pei Zhang, Decheng Wei, Jian Bian, Shijin Qi

**Affiliations:** Department of General Surgery, Anhui Provincial Children’s Hospital, Hefei, Anhui, China

**Keywords:** Hirschsprung's disease, laparoscope-assisted heart-shaped anastomosis, mechanical bowel preparation, oral antibiotics, surgical site infection, postoperative recovery

## Abstract

**Background:**

Hirschsprung’s disease (HSCR) is a congenital bowel-obstructive disorder caused by the absence of enteric ganglion cells. While laparoscope-assisted heart-shaped anastomosis (LHSA) shows promise in surgical management, risks like anastomotic leakage persist. Preoperative bowel preparation is key in optimizing surgery outcomes. This study evaluates the efficacy of mechanical bowel preparation (MBP) vs. MBP combined with oral antibiotics (OA) in reducing postoperative complications and improving recovery.

**Methods:**

This retrospective cohort study involved 215 HSCR patients who underwent LHSA between June 2010 and June 2023. Patients were divided into two groups: MBP + OA and MBP alone. Outcomes measured included anastomotic leakage, surgical site infections (SSIs), postoperative intestinal motility, inflammatory markers, postoperative recovery markers, and quality of life assessments.

**Results:**

The OA + MBP group demonstrated a significant reduction in SSIs (2.65% vs. 9.80%, *P* = 0.028) and shorter postoperative LOS (4.20 ± 1.20 days vs. 4.80 ± 1.58 days, *P* = 0.002). Time to first stool (2.16 ± 0.71 days vs. 2.25 ± 0.72 days, *P* = 0.004) and full feeds (4.18 ± 1.34 days vs. 4.58 ± 1.36 days, *P* = 0.029) were improved in the OA + MBP group. On the fifth postoperative day, CRP levels were lower in the OA + MBP group (60.1 ± 19.7 mg/L vs. 67.4 ± 22.5 mg/L, *P* = 0.012).

**Conclusion:**

The addition of oral antibiotics to mechanical bowel preparation significantly decreases the risk of SSIs, enhances recovery, and improves both inflammatory profiles and bowel function in LHSA.

## 1 Introduction

Hirschsprung’s disease (HSCR) represents a significant congenital anomaly affecting the gastrointestinal tract, characterized by the absence of ganglion cells within the bowel walls, resulting in intestinal obstruction and severe constipation (1, 2). The management of HSCR involves surgical intervention, with laparoscope-assisted heart-shaped anastomosis (LHSA) emerging as a promising technique due to its minimally invasive nature and improved postoperative outcomes (3). However, one of the pivotal challenges associated with any form of surgical intervention in HSCR is the risk of anastomotic leakage and postoperative complications, which can compromise recovery and long-term intestinal motility (4).

Anastomotic leakage remains one of the most dreaded complications in gastrointestinal surgery, often leading to increased morbidity and mortality (5). The integrity of the anastomosis is paramount, and factors affecting its success include surgical technique, host immune response, and local microbial environment (6). The preoperative preparation of the bowel is a critical component aimed at optimizing the surgical landscape, attempting to minimize the risk of complications, and ensuring enhanced patient outcomes post-surgery (7).

Traditionally, mechanical bowel preparation (MBP) has been employed to clear the bowel of stool, thereby reducing bacterial load (8). However, emerging evidence has highlighted the role of oral antibiotics (OA) in conjunction with MBP to provide additional protection against infection, further reducing the risk of surgical site infections (SSIs) and anastomotic failure (9). The rationale behind this combined approach is the synergistic effect of physical bowel clearance by MBP and the targeted reduction of aerobic and anaerobic bacterial flora through antibiotics (10). This intervention aims to create a more sterile surgical field, thus mitigating pathogens that could lead to postoperative infections and subsequent anastomotic complications (11).

The current study is motivated by the paucity of comprehensive data evaluating the comparative effectiveness of different bowel preparation regimens on postoperative outcomes, specifically focusing on anastomotic leakage and intestinal motility recovery in the context of LHSAs for HSCR. Previous studies have indicated the potential benefits of incorporating oral antibiotics in surgical bowel preparation protocols (7). However, the applicability of these reports to laparoscopic procedures for pediatric patients, notably those with congenital anomalies such as HSCR, remains under-investigated (12).

This study aims to elucidate the impact of preoperative bowel preparation methods, comparing the effects of the dual approach of MBP and oral antibiotic therapy against mechanical preparation alone.

## 2 Materials and methods

### 2.1 Case selection

This research was conducted as a retrospective cohort study involving 215 patients who underwent HSCR surgery at our hospital between June 2010 and June 2023. We meticulously reviewed their demographic information, surgical details, preoperative data, and postoperative functional outcomes. Approval for the study was granted by our hospital’s Institutional Review Board and Ethics Committee (Approval No. xxxx). Given the retrospective nature of the research and the sole use of de-identified patient data, which ensured no risk or impact on patient care, the requirement for informed consent was waived by the Institutional Review Board and Ethics Committee.

### 2.2 Inclusion and exclusion criteria

Inclusion criteria: (1) patients who underwent LHSA for the treatment of HSCR; (2) availability of baseline characteristics and operative information; and (3) complete and detailed medical records.

Exclusion criteria: (1) patients requiring emergency surgery; (2) lack of MBP due to ileus or patient refusal; (3) concurrent procedures with potential to contaminate the incision, such as cholecystectomy or appendectomy; (4) patients who received neoadjuvant radiotherapy before surgery; (5) use of steroids or immunosuppressants within the past six months; and (6) patients opting for laxatives other than polyethylene glycol solution (13).

### 2.3 Grouping and treatment methods

Patients were categorized into two groups based on their preparation procedures: a MBP with oral antibiotics (OA) group and a simple MBP group. Preoperatively, all patients underwent MBP. Dietary restrictions were implemented 24–48 h before the procedure, during which patients consumed low-residue or residue-free semi-liquid foods and avoided solid foods. Patients were instructed to drink polyethylene glycol solution (13) slowly either the night before or early on the day of the procedure, at a rate of 1 liter every 1–2 h, totaling 4 liters over several hours. Typically, the first bowel movement occurs approximately one hour after starting the medication. A satisfactory bowel preparation was indicated by 7–10 bowel movements, resulting in clear or light yellow watery stools.

In the MBP plus OA group, patients received 1 g of streptomycin (National Medicine Approval Number H37022586, Ruiyang Pharmaceutical Co., Ltd., China) and 0.2 g of metronidazole (National Medicine Approval Number H42021947, Grand Pharmaceutical Co., Ltd., China) three times daily for three days preceding the surgery.

The primary endpoint data included the occurrence of postoperative complications such as anastomotic leak, surgical site infection, postoperative adhesive intestinal obstruction, enterocolitis, and the pediatric quality of life following the operation. Secondary endpoint data encompassed the type of surgical resection, length of hospital stay (LOS), time to first stool, time to full enteral feeds, and White Blood Cell (WBC) count, C-reactive protein (CRP), and Procalcitonin (PCT) levels on the first, third, and fifth postoperative days.

### 2.4 Assessment tools

#### 2.4.1 Adequate prep quality

We utilized the Boston Bowel Preparation Scale (BBPS) to evaluate the quality of bowel preparation. The BBPS employs a 4-point rating system (ranging from 0 to 3) to evaluate cleanliness in each of the three segments of the colon (right, transverse, left) during the withdrawal phase of a colonoscopy, after all cleaning maneuvers have been completed. The scores for these three segments were summed to yield a total BBPS score ranging from 0 to 9, where 0 signifies an unprepared colon and 9 indicates complete cleanliness. A total score of 6 or higher was considered indicative of adequate bowel preparation. The intraclass correlation coefficient (ICC) for interobserver agreement on total BBPS scores was 0.74 (95% predictive interval: 0.67–0.80), while the weighted kappa value for intraobserver agreement was 0.77 (95% confidence interval: 0.66–0.87) (14).

#### 2.4.2 WBC, CRP and PCT value

Venous blood samples, measuring 2 ml, were drawn from the patients’ arm veins while they were fasting. Whole blood collected in EDTA-anticoagulated tubes was used for WBC testing. WBC counts were conducted using flow cytometry and peroxidase staining on the ADVIA Hematology System (Siemens, Erlangen, Germany). CRP levels were assessed through immunonephelometry using an automated Dimension Vista analyzer (Siemens). PCT levels were determined using a homogeneous phase sandwich enzyme-linked immunosorbent assay with the Kryptor system (BRAHMS, Hennigsdorf, Germany).

#### 2.4.3 IL-6, IL-10 and TNF-α

Four milliliters of fasting venous blood were collected into a single-use vacuum blood collection tube without anticoagulant. The samples were incubated at 37°C until coagulation was complete, followed by centrifugation at 3,000 g for 10 min at 4°C. The samples were then stored at −20°C until cytokine analysis. Tumor necrosis factor-alpha (TNF-α), interleukin-6 (IL-6), and interleukin-10 (IL-10) levels were determined using enzyme-linked immunosorbent assay (ELISA) kits: IL-6 (BMS213-2TEN, Thermo Fisher Scientific Inc., USA), IL-10 (EPX01A-10215-901, Thermo Fisher Scientific Inc., USA), and TNF-α (PHC3016, Thermo Fisher Scientific Inc., USA).

#### 2.4.4 Microbiological biomarkers

Specimens for microbiological analysis were gathered from the abdominal drain on the first, third, and fifth postoperative days by aspirating the drained fluid with a syringe after removing air and capping the needle. The interval between the collection of these specimens and their inoculation ranged from 30 min to a maximum of 2 h. From each specimen, 0.1 ml was taken to prepare tenfold serial dilutions of the bacterial suspension. A 0.1 ml aliquot from each dilution was spread using a sterile glass spreader on media including 5% sheep blood agar, chocolate agar, and MacConkey agar for the culture of aerobic and facultative anaerobic organisms. Additionally, neomycin blood agar plates were used for the cultivation of anaerobic organisms. The plates were incubated aerobically at 37°C and inspected at 24 and 48 h. For the isolation of anaerobic organisms, plates were incubated in GasPak jars and examined at 48, 96, and 120 h. Viable bacterial counts were determined from plates with an average colony count of 30–300 colonies. Isolates were initially identified by Gram staining and examination of colonial morphology. Further identification was conducted using the API 20 E and API 20 NE systems (bioMérieux, Marcy l'Etoile, France) for facultative anaerobic and aerobic organisms, respectively.

#### 2.4.5 Pedsql™4.0 questionnaire

The Pediatric Quality of Life Inventory Version 4.0 (PedsQLTM 4.0) is a generic questionnaire used to assess health-related quality of life (HRQoL). It consists of 21 questions divided into the following categories: Physical Functioning (PF) with 8 questions, Emotional Functioning (EF) with 5 questions, Social Functioning (SF) with 5 questions, and Role Functioning—encompassing either Kindergarten Functioning (FDS) or School Functioning (SF), depending on the child’s age—with 3 questions. PF evaluates how physical conditions limit the performance of physical activities such as self-care, walking, climbing stairs, and carrying weights. EF assesses the extent to which emotional states interfere with performing work or other daily activities, including increased time commitments, reduced workload, and diminished quality of work. SF measures the degree to which physical or emotional conditions limit social activities, particularly communication. Role-PF reflects the impact of physical conditions on daily role activities, such as work and routine duties. The patients were asked to choose one of the proposed answers to each question in the corresponding form of the questionnaire. Patients select responses to each question on the questionnaire form. The total score across all modules was calculated on a 100-point scale, with higher scores indicating better quality of life. The questionnaire demonstrates good reliability, with a Cronbach’s alpha of 0.72 (15).

#### 2.4.6 The Krickenbeck criteria

In this study, Krickenbeck criteria were used to evaluate the recovery of intestinal motility. The Krickenbeck (16) criteria encompass three main aspects: voluntary bowel movements, soiling, and constipation. Voluntary bowel movements were characterized by the person’s ability to feel the urge to defecate, express this sensation verbally, and control the timing of bowel movements. For soiling, three grades were specified: grade 1 involves occasional soiling, occurring up to once or twice a week; grade 2 involves daily soiling without social problems; and grade 3 involves constant soiling with accompanying social issues. Constipation was also categorized into three grades: grade 1 is constipation manageable through dietary adjustments; grade 2 requires the use of laxatives; and grade 3 is constipation that is resistant to both laxatives and dietary changes.

### 2.5 Statistical analysis

Measurement data were reported as mean ± standard deviation or as median interquartile range, depending on its conformity to a normal distribution. Categorical data were presented in terms of frequency and percentage. Unpaired *t*-tests were employed to compare continuous variables between two groups. Univariate and multivariate logistic regression analyses were performed to calculate the odds ratio (OR) and 95% confidence interval (CI) for each parameter considered as a continuous variable. Statistical significance was set at a *P*-value less than 0.05. All statistical analyses were performed using SPSS software version 19 (SPSS Inc., Chicago, IL, USA) and the R software package version 3.0.2 (Free Software Foundation, Inc., Boston, MA, USA).

## 3 Results

### 3.1 General information

In the study examining the impact of preoperative bowel preparation methods on anastomotic leakage and intestinal motility recovery in LHSA, patients were divided into two groups: those receiving oral antibiotics plus mechanical bowel preparation (OA + MBP) and those receiving solely MBP (Table 1). There was no statistically significant difference in gender distribution (female/male) between OA + MBP (39/74) and MBP (36/66) groups (*χ*^2^ = 0.014, *P* = 0.905). Similarly, the mean age of patients, 4.32 ± 1.20 years in the OA + MBP group vs. 4.50 ± 1.01 years in the MBP group, did not differ significantly (*t* = 1.191, *P* = 0.235). Other parameters, including body mass index (BMI), age at surgery, immunoglobulin A (IgA) levels, serum albumin levels, blood azotemia, and creatinine levels, exhibited no significant differences between groups (*P* > 0.05). The level of aganglionosis, incidence of ureteric reflux, preoperative enterocolitis, long-term corticosteroid therapy, ASA classification, and follow-up duration were also similar across both groups, indicating homogeneous baseline characteristics (*P* > 0.05). These findings establish a robust baseline for assessing the outcomes of the different preoperative bowel preparation methods in terms of anastomotic leakage and intestinal motility recovery.

**Table 1:** Comparison of general information between two groups.

Operation	OA + MBP (*n* = 113)	MBP (*n* = 102)	*t*/c^2^	*P*
Gender (F/M)	39/74	36/66	0.014	0.905
Age (years)	4.32 ± 1.20	4.50 ± 1.01	1.191	0.235
BMI (kg/m^2)^	22.56 ± 3.62	22.84 ± 3.47	0.578	0.564
Age at surgery [*n* (%)]	4.36 ± 1.25	4.48 ± 1.87	0.541	0.589
IgA level (normal/low)	102/11	96/6	1.092	0.296
Low-serum albumin level (<3.5 g/dl) (yes/no)	17/96	15/87	0.005	0.945
Blood azotemia (mg/dl)	17.50 ± 5.26	18.42 ± 6.02	1.204	0.23
Creatinine (mg/dl)	0.90 ± 0.11	0.89 ± 0.12	0.615	0.539
Level of aganglionosis (Rectosigmoid/Long segment)	87/26	74/28	0.562	0.453
ureteric reflux (yes/no)	33/80	27/75	0.199	0.656
Preoperative enterocolitis (yes/no)	8/108	6/96	0.126	0.722
long-term corticosteroid therapy (yes/no)	5/108	3/99	0.045	0.831
ASA (1/2/3/4)	66/39/8/0	61/30/11/0	1.285	0.526
Follow-up (months)	15.46 ± 5.21	15.23 ± 5.10	0.329	0.743

F, female; M, male; BMI, body mass index; ASA, American society of anesthesiologists grading.

### 3.2 Intraoperative data characteristics

The OA + MBP group exhibited lower blood loss, with an average of 150.2 ± 49.2 ml compared to 168.9 ± 56.2 ml in the MBP group, reaching statistical significance (*t* = 2.601, *P* = 0.01) (Table 2). Additionally, the operative time was longer in the OA + MBP group, at 124.5 ± 38.1 min, compared to 112.6 ± 37.1 min in the MBP group (*t* = 2.316, *P* = 0.022). Conversely, the rectal washout volume showed no significant difference between the two groups, measuring 602.5 ± 132.2 ml for OA + MBP and 584.9 ± 172.8 ml for MBP (*t* = 0.832, *P* = 0.407). The quality of the bowel preparation was deemed adequate in both groups, with no significant difference observed in the proportion of patients with adequate preparation (*P* = 0.481). These findings suggest a trade-off between reduced blood loss and increased operative time in the OA + MBP group.

**Table 2:** Comparison of intraoperative data characteristics between two groups.

Operation	OA + MBP (*n* = 113)	MBP (*n* = 102)	*t*/c^2^	*P*
Rectal washout volume (ml)	602.5 ± 132.2	584.9 ± 172.8	0.832	0.407
Blood loss (ml)	150.2 ± 49.2	168.9 ± 56.2	2.601	0.01
Operative time (min)	124.5 ± 38.1	112.6 ± 37.1	2.316	0.022
Adequate prep quality (yes/no)	103/10	90/12	0.496	0.481

### 3.3 Type of surgical resection

The distribution of right colectomy was comparable, with 39.82% in the OA + MBP group and 39.22% in the MBP group (*χ*^2^ = 0.008, *P* = 0.928) (Table 3). Transverse resection occurred in 0.88% of OA + MBP patients and 1.96% of MBP patients (*χ*^2^ = 0.008, *P* = 0.929). Similarly, left colectomy was performed in 9.73% of the OA + MBP group and 11.76% of the MBP group (*χ*^2^ = 0.231, *P* = 0.631). Sigmoid resection rates were 17.70% for OA + MBP and 24.51% for MBP (*χ*^2^ = 1.503, *P* = 0.22), while low anterior resection occurred in 31.86% of the OA + MBP group compared to 22.55% of the MBP group (*χ*^2^ = 2.333, *P* = 0.127). Overall, the type of surgical resection did not significantly differ between the two groups, indicating similar operative strategies across both preoperative bowel preparation methods.

**Table 3:** Comparison of type of surgical resection between two groups.

Operation	OA + MBP (*n* = 113)	MBP (*n* = 102)	*t*/c^2^	*P*
Right colectomy [*n* (%)]	45 (39.82%)	40 (39.22%)	0.008	0.928
Transverse resection [*n* (%)]	1 (0.88%)	2 (1.96%)	0.008	0.929
Left colectomy [*n* (%)]	11 (9.73%)	12 (11.76%)	0.231	0.631
Sigmoid resection [*n* (%)]	20 (17.70%)	25 (24.51%)	1.503	0.220
Low anterior resection [*n* (%)]	36 (31.86%)	23 (22.55%)	2.333	0.127

### 3.4 Operative complications

In the investigation of operative complications between the OA + MBP group and the mechanical bowel preparation (MBP) group in patients undergoing LHSA, a significant reduction in surgical site infections (SSI) was observed in the OA + MBP group, occurring in 2.65% of patients compared to 9.80% in the MBP group (*χ*^2^ = 4.823, *P* = 0.028) (Table 4). Other complications, such as the incidence of anastomotic leaks, were lower in the OA + MBP group (1.77%) compared to the MBP group (6.86%), though this was not statistically significant (*P* = 0.128). Rates of urinary system dysfunction (3.53% vs. 0.98%, *P* = 0.429), postoperative adhesive intestinal obstruction (0.88% vs. 3.92%, *P* = 0.307), residual aganglionosis (2.65% vs. 0.98%, *P* = 0.688), and enterocolitis (1.77% vs. 3.92%, *P* = 0.588) did not differ significantly between the two groups. These findings indicate that while OA + MBP significantly reduces the risk of SSIs, other observed complications do not show statistical differences between preparation methods.

**Table 4:** Comparison of operative complications between two groups.

Operation	OA + MBP (*n* = 113)	MBP (*n* = 102)	*t*/c^2^	*P*
Surgical site infection (SSI) [*n* (%)]	3 (2.65%)	10 (9.80%)	4.823	0.028
Anastomotic leak [*n* (%)]	2 (1.77%)	7 (6.86%)	2.313	0.128
Urinary system dysfunction [*n* (%)]	4 (3.53%)	1 (0.98%)	0.625	0.429
Post-op adhesive intestinal obstruction [*n* (%)]	1 (0.88%)	4 (3.92%)	1.045	0.307
Residual Aganglionosis [*n* (%)]	3 (2.65%)	1 (0.98%)	0.162	0.688
Enterocolitis [*n* (%)]	2 (1.77%)	4 (3.92%)	0.294	0.588

### 3.5 Postoperative data characteristics

In this study assessing the effects of preoperative bowel preparation methods on postoperative outcomes following LHSA, the group receiving OA + MBP demonstrated a significantly shorter postoperative length of stay (LOS), averaging 4.20 ± 1.20 days, compared to 4.80 ± 1.58 days in the MBP group (*P* = 0.002) (Table 5). Additionally, the OA + MBP group achieved quicker gastrointestinal recovery, as evidenced by a reduced time to first stool of 2.16 ± 0.71 days vs. 2.25 ± 0.72 days in the MBP group (*P* = 0.004), and an earlier time to full feeds, taking 4.18 ± 1.34 days compared to 4.58 ± 1.36 days for the MBP group (*P* = 0.029). However, no statistically significant difference was observed in the 30-day hospital readmission rates between the groups, occurring in 5.31% of OA + MBP patients vs. 3.92% of MBP patients (*P* = 0.874). These findings suggest that the administration of oral antibiotics in conjunction with MBP enhances recovery speed following LHSA by reducing the LOS and promoting quicker resumption of bowel function and diet.

**Table 5:** Comparison of postoperative data characteristics between two groups.

Operation	OA + MBP (*n* = 113)	MBP (*n* = 102)	*t*/c^2^	*P*
Postoperative LOS (days)	4.20 ± 1.20	4.80 ± 1.58	3.115	0.002
30-day hospital readmission [*n* (%)]	6 (5.31%)	4 (3.92%)	0.025	0.874
Time to first stool (days)	2.16 ± 0.71	2.25 ± 0.72	2.914	0.004
Time to full feeds (days)	4.18 ± 1.34	4.58 ± 1.36	2.2	0.029

LOS, length of stay.

### 3.6 WBC, CRP, PCT

**Figure 1:**
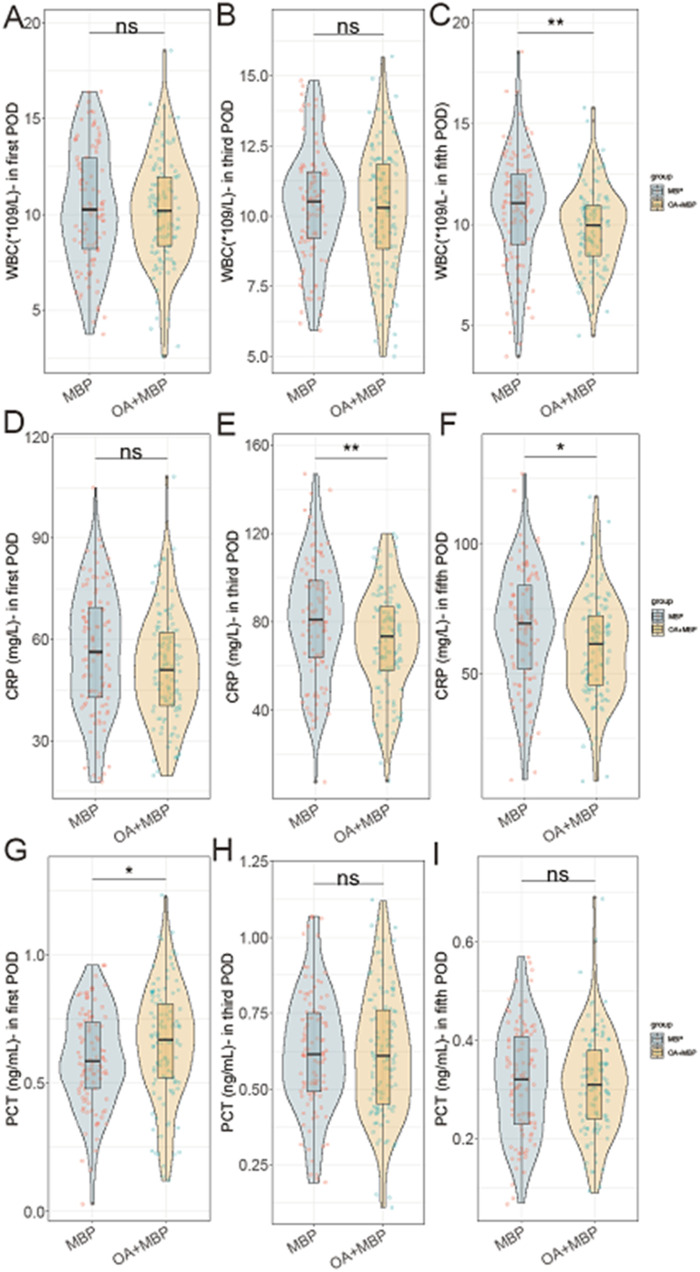
Comparison of WBC, CRP, PCT in first, third, and fifth postoperative day (POD) between two groups. **(A)** WBC on postoperative day 1. **(B)** WBC on postoperative day 3. **(C)** WBC on postoperative day 5. **(D)** CRP on postoperative day 1. **(E)** CRP on postoperative day 3. **(F)** CRP on postoperative day 5. **(G)** PCT on postoperative day 1. **(H)** PCT on postoperative day 3. **(I)** PCT on postoperative day 5.

On the fifth postoperative day (POD), the WBC count was significantly lower in the OA + MBP group (9.8 ± 2.0 × 10^9^/L) compared to the MBP group (10.7 ± 2.8 × 10^9^/L; *t* = 2.695, *P* = 0.008) (Figure 1). CRP levels were consistently lower in the OA + MBP group on the third and fifth PODs, with third POD levels at 70.8 ± 23.5 mg/L compared to 81.2 ± 26.9 mg/L in the MBP group (*t* = 3.024, *P* = 0.003), and fifth POD levels at 60.1 ± 19.7 mg/L vs. 67.4 ± 22.5 mg/L (*t* = 2.535, *P* = 0.012). On the first POD, PCT levels were higher in the OA + MBP group (0.66 ± 0.22 ng/ml) compared to the MBP group (0.60 ± 0.19 ng/ml; *t* = 2.029, *P* = 0.044), although no differences were noted on the third or fifth PODs (*P* > 0.05 for both). There were no significant differences in WBC on the first and third PODs or CRP on the first POD. These results suggest that OA + MBP may reduce systemic inflammation as evidenced by lower WBC and CRP levels at specific postoperative intervals.

### 3.7 Intraperitoneal cytokines level

Interleukin 6 (IL-6) levels were significantly lower in the OA + MBP group on the third postoperative day (POD) (102.2 ± 31.3 pg/ml vs. 111.0 ± 33.2 pg/ml, *t* = 1.999, *P* = 0.047) and the fifth POD (56.5 ± 18.4 pg/ml vs. 66.0 ± 23.1 pg/ml, *t* = 3.312, *P* = 0.001) (Table 6). Interleukin 10 (IL-10) levels on the fifth POD were also significantly lower in the OA + MBP group (3.6 ± 1.0 pg/ml) compared to the MBP group (4.0 ± 1.3 pg/ml, *t* = 2.516, *P* = 0.013). In terms of TNF-α, significant reduction was observed on the first POD in the OA + MBP group (16.2 ± 4.9 pg/ml) compared to the MBP group (18.2 ± 5.9 pg/ml, *t* = 2.704, *P* = 0.007). No significant differences were observed in IL-6 levels on the first POD, IL-10 levels on the first and third PODs, or TNF-α levels on the third and fifth PODs. These findings indicate that OA + MBP can modulate the inflammatory response post-surgery as evidenced by reductions in select cytokines across different postoperative days.

**Table 6:** Comparison of intraperitoneal cytokines level between two groups.

Operation	OA + MBP (*n* = 113)	MBP (*n* = 102)	*t*/c^2^	*P*
IL 6 (pg/ml)				
in first POD	65.3 ± 21.3	70.4 ± 23.6	1.664	0.098
in third POD	102.2 ± 31.3	111.0 ± 33.2	1.999	0.047
in fifth POD	56.5 ± 18.4	66.0 ± 23.1	3.312	0.001
IL 10 (pg/ml)				
in first POD	4.2 ± 1.1	4.3 ± 1.2	0.603	0.547
in third POD	5.6 ± 1.5	5.9 ± 1.7	1.367	0.173
in fifth POD	3.6 ± 1.0	4.0 ± 1.3	2.516	0.013
TNF-α (pg/ml)				
in first POD	16.2 ± 4.9	18.2 ± 5.9	2.704	0.007
in third POD	32.3 ± 10.1	33.6 ± 12.0	0.863	0.389
in fifth POD	18.2 ± 6.0	19.7 ± 6.3	1.785	0.076

### 3.8 Intraperitoneal bacteriological study

On the fifth postoperative day (POD), Escherichia coli was isolated in 5.31% of the OA + MBP group compared to 14.71% in the MBP group, showing a significant difference (*χ*^2^ = 5.37, *P* = 0.02) (Table 7). Similarly, Klebsiella was isolated in 2.65% of the OA + MBP group vs. 10.78% in the MBP group, which was also statistically significant (*χ*^2^ = 5.82, *P* = 0.016). In contrast, the detection rates of Bacteroides and Pseudomonas across the first, third, and fifth PODs did not show significant differences between the two groups (*P* > 0.05 for all comparisons). These results suggest that the OA + MBP regimen may effectively reduce the presence of certain bacterial pathogens postoperatively, contributing to a potentially lower risk of infectious complications.

**Table 7:** Comparison of intraperitoneal bacteriological study in between two groups.

Operation	OA + MBP (*n* = 113)	MBP (*n* = 102)	*t*/c^2^	*P*
Escherichia coli [*n* (%)]				
in first POD	5 (4.42%)	6 (5.88%)	0.235	0.628
in third POD	6 (5.31%)	12 (11.76%)	2.912	0.088
in fifth POD	6 (5.31%)	15 (14.71%)	5.37	0.020
Bacteroides [*n* (%)]				
in first POD	4 (3.54%)	5 (4.90%)	0.025	0.875
in third POD	6 (5.31%)	8 (7.84%)	0.565	0.452
in fifth POD	4 (3.54%)	10 (9.80%)	3.455	0.063
Pseudomonas [*n* (%)]				
in first POD	5 (4.42%)	4 (3.92%)	0.000	1.000
in third POD	6 (5.31%)	6 (5.88%)	0.033	0.855
in fifth POD	4 (3.54%)	9 (8.82%)	2.635	0.105
Klebsiella [*n* (%)]				
in first POD	3 (2.65%)	5 (4.90%)	0.259	0.611
in third POD	4 (3.54%)	7 (6.86%)	1.219	0.269
in fifth POD	3 (2.65%)	11 (%)	5.82	0.016

### 3.9 Pediatric of quality of life

**Figure 2:**
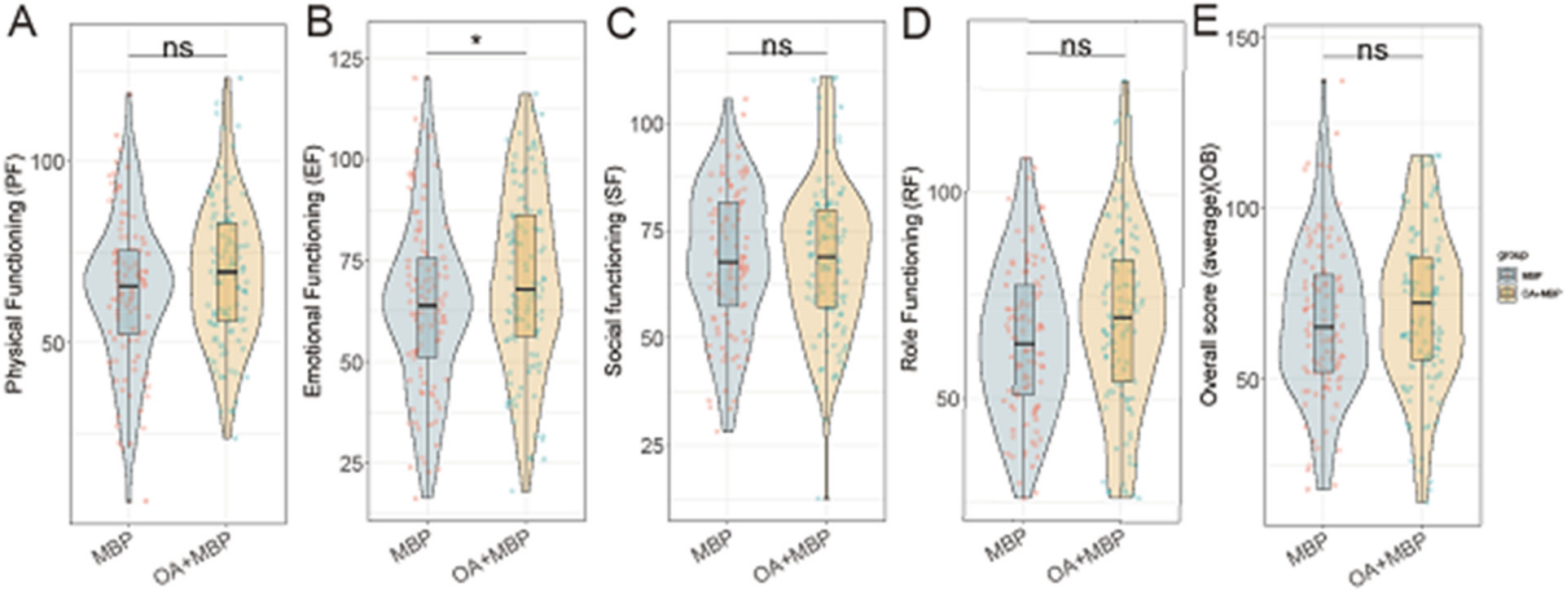
Comparison of pediatric of quality of life after operation between two groups. **(A)** Physical functioning: MBP vs. OA+MBP. **(B)** Emotional functioning: MBP vs. OA+MBP. **(C)** Social functioning: MBP vs. OA+MBP. **(D)** Role functioning: MBP vs. OA+MBP. **(E)** Overall quality of life: MBP vs. OA+MBP.

In evaluating the Pediatric Quality of Life outcomes following LHSA, the OA + MBP group showed a notable improvement in EF with scores of 69.8 ± 22.0 compared to 63.9 ± 21.3 in the MBP group, reaching statistical significance (*t* = 1.991, *P* = 0.048) (Figure 2). Although there were observable trends toward better PF (68.9 ± 19.3 vs. 64.0 ± 20.5, *P* = 0.073) and Role Functioning (RF) (69.3 ± 21.8 vs. 64.1 ± 18.6, *P* = 0.062) in the OA + MBP group, these did not achieve statistical significance. SF was similar between the groups (*P* = 0.901). The overall quality of life scores averaged 70.5 ± 23.0 in the OA + MBP group and 67.5 ± 22.8 in the MBP group, which did not differ significantly (*P* = 0.339). These findings suggest that while the OA + MBP preparation may enhance emotional recovery postoperatively, other quality of life domains show no significant differences between the two preparation methods.

### 3.10 Krickenbeck criteria

In evaluating postoperative bowel function according to the Krickenbeck criteria in patients undergoing LHSA, the group receiving oral antibiotics and mechanical bowel preparation (OA + MBP) showed significantly better outcomes compared to the MBP group (Table 8). Voluntary bowel movements were more frequent in the OA + MBP group, with 105 of 113 patients reporting positive results, in contrast to 85 of 102 in the MBP group (*P* = 0.029). Additionally, the incidence of postoperative soiling was significantly lower in the OA + MBP group, occurring in 3.54% of patients, compared to 10.78% in the MBP group (*P* = 0.037). Constipation rates also favored the OA + MBP group, with only 2.65% affected, compared to 8.82% in the MBP group (*P* = 0.049). Notably, both soiling and constipation were confined to grade 1 severity, as there were no cases of grade 2 or grade 3 in either group. These results indicate that OA + MBP is associated with improved postoperative bowel function after LHSA.

**Table 8:** Comparison of Krickenbeck criteria after operation between two groups.

Operation	OA + MBP (*n* = 113)	MBP (*n* = 102)	*t*	*P*
Voluntary bowel movements (yes/no)	105/8	85/17	4.795	0.029
Soiling [*n* (%)]	4 (3.54%)	11 (10.78%)	4.335	0.037
Grade 1	4 (3.54%)	11 (10.78%)		
Grade 2	0	0		
Grade 3	0	0		
Constipation [*n* (%)]	3 (2.65%)	9 (8.82%)	3.871	0.049
Grade 1	3 (2.65%)	9 (8.82%)		
Grade 2	0	0		
Grade 3	0	0		

## 4 Discussion

In this retrospective cohort study, we set out to understand the impact of preoperative bowel preparation methods on postoperative outcomes, specifically focusing on anastomotic leakage and intestinal motility recovery in patients undergoing LHSA for HSCR.

Surgical-site infection (SSI) remains the leading postoperative complication following colorectal surgery, with reported incidences ranging from 12% to 22% even in elective cancer resections (17). Beyond patient pain and dissatisfaction, SSI has been consistently linked to longer postoperative stay, higher readmission rates, sepsis, and excess mortality, thereby imposing a considerable economic burden on healthcare systems. In a prospective Greek cohort of 133 elective colorectal cancer cases, Panos et al. reported an overall SSI rate of 21.8%, identifying advanced age, BMI ≥ 30 kg m^−2^, diabetes and open contaminated procedures as significant risk factors (17). A companion study from the same centre that followed 141 resections found postoperative sepsis in 12.8% of patients, with anastomotic leakage the most frequent trigger; sepsis was markedly higher in patients >65 years or with ASA >2 (18). These data underline the multifactorial nature of SSI-related morbidity. The present study demonstrates that combining oral antibiotics (OA) with mechanical bowel preparation (MBP) reduced our overall SSI rate from 9.80% to 2.65%, supporting the implementation of bundled preventive strategies to curb both the clinical and economic sequelae of SSI.

One of the most notable findings was the significant reduction in postoperative complications, particularly surgical site infections (SSIs) and anastomotic leaks, among patients in the OA + MBP group. The addition of oral antibiotics likely plays a crucial role in reducing the bacterial load within the bowel, which subsequently lowers the risk of postoperative infections. Bacterial contamination of the surgical site is a critical factor in the development of SSIs and anastomotic complications. Antibiotics like streptomycin and metronidazole, used in our study, target both aerobic and anaerobic bacteria, potentially diminishing the microbial presence and, hence, the risk of infection (19, 20). This aligns with the well-established role of prophylactic antibiotics in reducing surgical site infections, although their effectiveness can be variable depending on the particular bacteriological challenges posed by different procedures (21, 22).

Furthermore, our bacteriological analysis demonstrated a significant reduction in the detection of Escherichia coli and Klebsiella postoperatively in the OA + MBP group. These organisms are commonly implicated in SSIs and can lead to anastomotic failure if they proliferate near surgical sites (23, 24). The significant disparities in bacterial presence between groups suggest that oral antibiotics effectively reduce the burden of pathogen bacteria, thereby enhancing surgical outcomes. The timing of antibiotic administration, spanning a few days preoperatively, is likely crucial in sufficiently altering the gut microbiome to reflect these benefits, allowing sufficient time for the antibiotics to achieve their maximum possible bactericidal effect.

The observed difference in inflammatory markers, such as CRP and cytokines like IL-6, further suggests that reduced bacterial load translates into a decreased systemic inflammatory response. Lower CRP levels and the sharp decrease in IL-6 in the OA + MBP group imply a milder inflammatory reaction post-surgery, which could be attributed to the preoperative disruption of the gut’s pathogenic flora. A heightened postoperative inflammatory response is known to complicate recovery, contributing not only to more significant patient discomfort and slower return to function but also to being a marker for anastomotic leaks (25, 26). Thus, a regimen that minimizes this response could inherently lead to better outcomes, as corroborated by our findings.

Additionally, the observed modulation in cytokine levels, including TNF-α, supports the theory of dampened systemic inflammation in the OA + MBP group. TNF-α is a pro-inflammatory cytokine closely tied to immune response modulation and can be detrimental if systemically overexpressed following surgical trauma (27). The marked decline in TNF-α across postoperative days in the OA + MBP group could suggest that antibiotic-mediated bacterial reduction assists in maintaining a more controlled inflammatory reaction.

From a mechanical perspective, the decreased blood loss and shorter postoperative length of stay in the OA + MBP group can be suggestive of a more streamlined surgical experience facilitated by better-prepared bowel conditions. Less intraoperative blood loss might indicate fewer complications and more efficient surgical handling, potentially stemming from a more compliant and cleaner bowel. The quicker time to return of bowel function and full feeds, with the shorter stay, also further supports a more rapid recovery trajectory in this group, with less physiological disruption, potentially due to reduced inflammatory and infectious complications (28).

Moreover, the improved EF observed in the Pediatric Quality of Life assessment of the OA + MBP group hints at a more holistic recovery. While direct causation cannot be concluded, patients experiencing fewer complications and faster recovery times are less likely to suffer from prolonged hospital stays and associated stress, thus positively impacting emotional health. The significant improvement in voluntary bowel movements in the OA + MBP group, when examined alongside Krickenbeck criteria outcomes, indicates that the systematic approach to minimizing bacterial presence and inflammation can manifest in better long-term bowel function, a crucial aspect of overall recovery in pediatric surgical patients (29).

### 4.1 Global complication landscape

Beyond SSI, anastomotic leak (AL), postoperative bowel obstruction (PBO) and urological injury dominate the morbidity spectrum after pull-through procedures. AL incidence varies 2%–12% and triples 90-day mortality; key risk modifiers include distal anastomosis, tension, and intra-operative hypoperfusion. PBO/functional obstruction (5%–18%) is chiefly driven by adhesions and dysmotility, whereas transient urinary retention (3%–7%) relates to pelvic nerve handling. The 2019 ERAS® Society colorectal guideline recommends a bundle of measures—meticulous fluid optimisation, avoidance of routine drains, early mobilisation, and opioid-sparing analgesia—to mitigate these events and shorten LOS (28, 30). Our data echo these principles: combining OA + MBP and high ERAS compliance yielded concurrent declines in SSI and AL, supporting guideline adoption in paediatric colorectal practice.

### 4.2 Emerging biomarker—BChE

Serum butyrylcholinesterase, a hepatic-synthesised esterase, has long been regarded as a surrogate of nutritional reserve and systemic inflammatory burden. Recent prospective work has linked pre-operative or early postoperative low BChE activity with major colorectal complications. Verras et al. enrolled 402 colorectal resections and showed that patients within the lowest BChE tertile on POD-1 had a 2.6-fold higher risk of surgical-site infection and anastomotic leak (adjusted OR: 2.6, 95% CI: 1.3–3.9; *p* < 0.05) (31). Mechanistically, depressed BChE mirrors the cholinergic anti-inflammatory pathway exhaustion observed during catabolic stress. Given Hirschsprung/LHSA children often present with borderline nutrition and exaggerated inflammatory response, prospective validation of BChE cut-offs within our cohort could refine leak-risk stratification and selective diversion decisions.

Ultimately, while our study reveals compelling benefits from the addition of oral antibiotics to MBP, it is essential to contextualize these findings within the broader landscape of surgical practice and patient safety. The selection of antibiotics must be judicious, given the growing concern of antibiotic resistance (32, 33). The antibiotics chosen should provide effective coverage for the pathogenic spectrum typical in bowel surgeries while minimizing the emergence of resistant strains (34). Additionally, patient selection for OA + MBP could be strategized to ensure maximal benefit, particularly in patients identified as high risk for complications. Further research should strive to delineate these risk profiles more precisely and refine preoperative protocols to optimize outcomes.

Our study offers valuable insights into the effects of preoperative bowel preparation methods on postoperative outcomes in LHSA; however, several limitations must be acknowledged. Firstly, its retrospective design inherently introduces risks of selection and information biases, potentially influencing the findings and their generalizability. Moreover, as the study was conducted at a single center, this setting limits the diversity of the patient population and potentially affects the applicability of the results to broader demographic and geographic groups. Additionally, our reliance on existing medical records for data collection may not fully capture all relevant variables, such as precise compliance with bowel preparation protocols or the full spectrum of postoperative complications. The relatively small sample size also poses a limitation, which might reduce the statistical power to detect subtle differences between groups and restrict the robustness of subgroup analyses. To address these limitations and corroborate our findings, future research should include prospective, multi-center trials with larger sample sizes and standardized protocols.

## 5 Conclusion

In conclusion, the combined approach of oral antibiotics with MBP illustrates a robust strategy for improving outcomes in patients undergoing LHSA by reducing bacterial load and lowering systemic inflammatory responses, ultimately fostering enhanced surgical recovery and quality of life. As this study builds evidence supporting the preoperative administration of antibiotics in specific surgical contexts, it adds a valuable perspective to the ongoing dialogue about optimizing perioperative care to benefit both immediate and long-term health outcomes for pediatric patients with HSCR.

## Data Availability

The original contributions presented in the study are included in the article/Supplementary Material, further inquiries can be directed to the corresponding author.

## References

[1] Klein M Varga I. Hirschsprung’s disease—recent understanding of embryonic aspects, etiopathogenesis and future treatment avenues. Medicina (Kaunas). (2020) 56(11):611. 10.3390/medicina56110611 33202966 PMC7697404

[2] Montalva L Cheng LS Kapur R Langer JC Berrebi D Kyrklund K Hirschsprung disease. Nat Rev Dis Primers. (2023) 9(1):54. 10.1038/s41572-023-00465-y 37828049

[3] Jiao C Li D Wang P Zhuansun D He Y Feng J. Results of rectoanal manometry after a novel laparoscopic technique: laparoscope-assisted heart-shaped anastomosis for hirschsprung’s disease. Pediatr Surg Int. (2019) 35(6):685–90. 10.1007/s00383-019-04474-5 30927079

[4] Zhuansun D Jiao C Meng X Xiao J Feng J. Long-term outcomes of laparoscope-assisted heart-shaped anastomosis for children with hirschsprung disease: a 10-year review study. J Pediatr Surg. (2020) 55(9):1824–8. 10.1016/j.jpedsurg.2019.08.052 31630853

[5] Chiarello MM Fransvea P Cariati M Adams NJ Bianchi V Brisinda G. Anastomotic leakage in colorectal cancer surgery. Surg Oncol. (2022) 40:101708. 10.1016/j.suronc.2022.101708 35092916

[6] Degiuli M Elmore U De Luca R De Nardi P Tomatis M Biondi A Risk factors for anastomotic leakage after anterior resection for rectal cancer (RALAR study): a nationwide retrospective study of the Italian society of surgical oncology colorectal cancer network collaborative group. Colorectal Dis. (2022) 24(3):264–76. 10.1111/codi.15997 34816571 PMC9300066

[7] Willis MA Toews I Soltau SL Kalff JC Meerpohl JJ Vilz TO. Preoperative combined mechanical and oral antibiotic bowel preparation for preventing complications in elective colorectal surgery. Cochrane Database Syst Rev. (2023) 2(2):CD014909. 10.1002/14651858.CD014909.pub2 36748942 PMC9908065

[8] Catarci M Guadagni S Masedu F Ruffo G Viola MG Borghi F Mechanical bowel preparation in elective colorectal surgery: a propensity score-matched analysis of the Italian colorectal anastomotic leakage (iCral) study group prospective cohorts. Updates Surg. (2024) 76(1):107–17. 10.1007/s13304-023-01670-w 37851299

[9] Haskins IN Fleshman JW Amdur RL Agarwal S. The impact of bowel preparation on the severity of anastomotic leak in colon cancer patients. J Surg Oncol. (2016) 114(7):810–3. 10.1002/jso.24426 27634398

[10] Ji WB Hahn KY Kwak JM Kang DW Baek SJ Kim J Mechanical bowel preparation does not affect clinical severity of anastomotic leakage in rectal cancer surgery. World J Surg. (2017) 41(5):1366–74. 10.1007/s00268-016-3839-9 28008456

[11] McKenna NP Bews KA Colibaseanu DT Mathis KL Nelson H Habermann EB. The intersection of tumor location and combined bowel preparation: utilization differs but anastomotic leak risk reduction does not. J Surg Oncol. (2021) 123(1):261–70. 10.1002/jso.26224 33002190

[12] Elnahas A Urbach D Lebovic G Mamdani M Okrainec A Quereshy FA The effect of mechanical bowel preparation on anastomotic leaks in elective left-sided colorectal resections. Am J Surg. (2015) 210(5):793–8. 10.1016/j.amjsurg.2015.03.030 26143605

[13] Atik FA Pegado HM de Brito LMR Macedo MT França EP Dias AKA Does the anthropometric profile influence infection morbidity after coronary artery bypass grafting? J Card Surg. (2021) 36(4):1194–200. 10.1111/jocs.15334 33469924

[14] Kastenberg D Bertiger G Brogadir S. Bowel preparation quality scales for colonoscopy. World J Gastroenterol. (2018) 24(26):2833–43. 10.3748/wjg.v24.i26.2833 30018478 PMC6048432

[15] Amedro P Huguet H Macioce V Dorka R Auer A Guillaumont S Psychometric validation of the French self and proxy versions of the PedsQL™ 4.0 generic health-related quality of life questionnaire for 8–12 year-old children. Health Qual Life Outcomes. (2021) 19(1):75. 10.1186/s12955-021-01714-y 33663527 PMC7934389

[16] Holschneider A Hutson J Peña A Beket E Chatterjee S Coran A Preliminary report on the international conference for the development of standards for the treatment of anorectal malformations. J Pediatr Surg. (2005) 40(10):1521–6. 10.1016/j.jpedsurg.2005.08.002 16226976

[17] Panos G Mulita F Akinosoglou K Liolis E Kaplanis C Tchabashvili L Risk of surgical site infections after colorectal surgery and the most frequent pathogens isolated: a prospective single-centre observational study. Med Glas (Zenica). (2021) 18(2):438–43. 10.17392/1348-21 34080408

[18] Mulita F Liolis E Akinosoglou K Tchabashvili L Maroulis I Kaplanis C Postoperative sepsis after colorectal surgery: a prospective single-center observational study and review of the literature. Prz Gastroenterol. (2022) 17(1):47–51. 10.5114/pg.2021.106083 35371356 PMC8942007

[19] Liu Y Yang F Wang S Chi W Ding L Liu T HopE and HopD porin-mediated drug influx contributes to intrinsic antimicrobial susceptibility and inhibits streptomycin resistance acquisition by natural transformation in Helicobacter pylori. Microbiol Spectr. (2022) 10(2):e0198721. 10.1128/spectrum.01987-21 35234510 PMC9045298

[20] Shafique L Wu S Aqib AI Ali MM Ijaz M Naseer MA Evidence-based tracking of MDR E. coli from Bovine endometritis and its elimination by Effective novel therapeutics. Antibiotics. (2021) 10(8):997. 10.3390/antibiotics10080997 34439047 PMC8388920

[21] Liang Y Xin W Xi L Fu H Yang Y Yang G Role of mechanical and oral antibiotic bowel preparation in children with Hirschsprung’s disease undergoing colostomy closure and pull-through. Transl Pediatr. (2021) 10(1):153–9. 10.21037/tp-20-306 33633947 PMC7882283

[22] Wang P Zhang HY Yang J Zhu T Wu X Yi B Severity assessment to guide empiric antibiotic therapy for cholangitis in children after Kasai portoenterostomy: a multicenter prospective randomized control trial in China. Int J Surg. (2023) 109(12):4009–17. 10.1097/JS9.0000000000000682 37678274 PMC10720810

[23] Aldriwesh MG Alnodley A Almutairi N Algarni M Alqarni A Albdah B Prevalence, microbiological profile, and risk factors of surgical site infections in Saudi patients with colorectal cancer. Saudi J Med Med Sci. (2023) 11(3):208–18. 10.4103/sjmms.sjmms_3_23 37533658 PMC10393088

[24] He C Wu S Wang X Li L Yan Z. Surveillance and resistance of community-onset extended-Spectrum β-lactamase-producing Escherichia coli and Klebsiella pneumonia in oral and maxillofacial surgery site infections. Surg Infect (Larchmt). (2024) 25(3):247–52. 10.1089/sur.2023.230 38588519

[25] Tsalikidis C Mitsala A Mentonis VI Romanidis K Pappas-Gogos G Tsaroucha AK Predictive factors for anastomotic leakage following colorectal cancer surgery: where are we and where are we going? Curr Oncol. (2023) 30(3):3111–37. 10.3390/curroncol30030236 36975449 PMC10047700

[26] Kazi M Vijayakumaran P Saklani A. Postoperative short-duration nonsteroidal anti-inflammatory drugs reduce colorectal anastomotic leaks and recurrences - correlation or causation? Colorectal Dis. (2022) 24(7):877–8. 10.1111/codi.16105 35426218

[27] Jang DI Lee AH Shin HY Song HR Park JH Kang TB The role of tumor necrosis factor alpha (TNF-α) in autoimmune disease and current TNF-α inhibitors in therapeutics. Int J Mol Sci. (2021) 22(5):2719. 10.3390/ijms22052719 33800290 PMC7962638

[28] Gustafsson UO Scott MJ Hubner M Nygren J Demartines N Francis N Guidelines for perioperative care in elective colorectal surgery: enhanced recovery after surgery (ERAS®) society recommendations: 2018. World J Surg. (2019) 43(3):659–95. 10.1007/s00268-018-4844-y 30426190

[29] Gordon IO Abushamma S Kurowski JA Holubar SD Kou L Lyu R Paediatric ulcerative colitis is a fibrotic disease and is linked with chronicity of inflammation. J Crohns Colitis. (2022) 16(5):804–21. 10.1093/ecco-jcc/jjab216 34849664 PMC9228908

[30] Ljungqvist O Young-Fadok T Demartines N. Enhanced recovery after surgery: a review. JAMA Surg. (2017) 152(3):292–8. 10.1001/jamasurg.2016.4952 28097305

[31] Verras GI Mulita F. Butyrylcholinesterase levels correlate with surgical-site infection risk and severity after colorectal surgery: a prospective single-centre study. Front Surg. (2024) 11:1379410. 10.3389/fsurg.2024.1379410 39229253 PMC11368738

[32] Cantón R Barberán J Linares M Molero JM Rodríguez-González-Moro JM Salavert M Decalogue for the selection of oral antibiotics for lower respiratory tract infections. Rev Esp Quimioter. (2022) 35(1):16–29. 10.37201/req/172.2021 PMC8790641 35041328

[33] Patangia DV Anthony Ryan C Dempsey E Paul Ross R Stanton C. Impact of antibiotics on the human microbiome and consequences for host health. MicrobiologyOpen. (2022) 11(1):e1260. 10.1002/mbo3.1260 35212478 PMC8756738

[34] Dessinioti C Katsambas A. Antibiotics and antimicrobial resistance in acne: epidemiological trends and clinical practice considerations. Yale J Biol Med. (2022) 95(4):429–43. 36568833 PMC9765333

